# Pullulan microbeads/Si-HPMC hydrogel injectable system for the sustained delivery of GDF-5 and TGF-β1: new insight into intervertebral disc regenerative medicine

**DOI:** 10.1080/10717544.2017.1340362

**Published:** 2017-06-23

**Authors:** Nina Henry, Johann Clouet, Audrey Fragale, Louise Griveau, Claire Chédeville, Joëlle Véziers, Pierre Weiss, Jean Le Bideau, Jérôme Guicheux, Catherine Le Visage

**Affiliations:** a INSERM, UMRS 1229, RMeS “Regenerative Medicine and Skeleton”, Team STEP “Skeletal Physiopathology and Joint Regenerative Medicine”, Nantes, France;; b Institut des Matériaux Jean Rouxel (IMN), Université de Nantes, CNRS, Nantes, France;; c UFR Odontologie, Université de Nantes, Nantes, France;; d CHU Nantes, PHU 11 Pharmacie, Pharmacie Centrale, Nantes, France;; e UFR Sciences Biologiques et Pharmaceutiques, Université de Nantes, Nantes, France;; f SC3M platform, UMS INSERM 016/CNRS 3556, SFR François Bonamy, Nantes, France;; g CHU Nantes, PHU 4 OTONN, Nantes, France;; h INSERM, UMRS 1229, RMeS “Regenerative Medicine and Skeleton”, Team REGOS “Regenerative Medicine of Bone Tissues”, Nantes, France

**Keywords:** Drug delivery, hydrogel, IVD, microcarriers, polysaccharide

## Abstract

Discogenic low back pain is considered a major health concern and no etiological treatments are today available to tackle this disease. To clinically address this issue at early stages, there is a rising interest in the stimulation of local cells by *in situ* injection of growth factors targeting intervertebral disc (IVD) degenerative process. Despite encouraging safety and tolerability results in clinic, growth factors efficacy may be further improved. To this end, the use of a delivery system allowing a sustained release, while protecting growth factors from degradation appears of particular interest. We propose herein the design of a new injectable biphasic system, based on the association of pullulan microbeads (PMBs) into a cellulose-based hydrogel (Si-HPMC), for the TGF-β1 and GDF-5 growth factors sustained delivery. We present for the first time the design and mechanical characterization of both the PMBs and the called biphasic system (PMBs/Si-HPMC). Their loading and release capacities were also studied and we were able to demonstrate a sustained release of both growth factors, for up to 28 days. Noteworthy, the growth factors biological activity on human cells was maintained. Altogether, these data suggest that this PMBs/Si-HPMC biphasic system may be a promising candidate for the development of an innovative bioactive delivery system for IVD regenerative medicine.

## Introduction

Intervertebral disc (IVD) is a fibrocartilaginous tissue located between each vertebrae, which plays a key role in the spine kinematics allowing trunk movement. Early after birth, this structure, and notably its central part [i.e. the *Nucleus pulposus* (NP)], initiates a degenerative process. This degenerative process is associated with a decrease of the number of resident cells, leading to an alteration of the extracellular matrix homeostasis and the emergence of a fibrosis, leading altogether to impaired IVD biomechanical functions (Colombier et al., 2014). Disc degenerative disease (DDD) is one of the leading cause of low back pain and is considered a major health concern (Manchikanti et al., 2009). Current therapies mainly aim at tackling low back pain using pharmacological treatments or surgical approaches (arthroplasty or spine fusion), notably for the most advanced debilitating DDD cases. Unfortunately, none of these strategies address the etiological cause of the disease. To clinically address this issue and reactivate the biological machinery, regenerative medicine approaches are considered with deep interest. With respect to the limited clinical translatability of cell-based regenerative medicine approaches, the use of biological factors targeting the degenerative process of IVD has recently been contemplated, notably growth factors able to stimulate NP cells (Whatley & Wen, 2012; Blanquer et al., 2014). In this context, we recently demonstrated the synergistic role of two growth factors, the transforming growth factor-β1 (TGF-β1) and the growth and differentiation factor-5 (GDF-5), in driving the *in vitro* differentiation of human adipose stromal cells (hASC) towards NP cells (Colombier et al., 2016). Interestingly, the culture of hASCs in a media enriched with these growth factors has been shown to induce the production of a specific extracellular matrix, rich in glycosaminoglycans. In addition, recent clinical trials have been conducted to investigate the effectiveness of the intradiscal injection of GDF-5 in patients with early DDD (NCT01124006, NCT00813813, NCT01182337, NCT01158924 on clinicaltrial.gov). Despite promising safety and tolerability, the clinical outcomes need further improvement before GDF-5 may enter the arsenal of routinely available therapeutics. Meanwhile, to improve the clinical efficacy of growth factors in IVD regenerative medicine and properly exploit their nucleopulpogenic potential, specific biomaterial-assisted drug delivery systems (DDS) allowing a sustained release, while protecting them from degradation, have been considered (Blanquer et al., 2014).

Various biomaterials have been investigated, including polysaccharides, that have raised a particular interest. Polysaccharides are naturally derived polymers obtained from renewable sources such as plants, animals or produced by fungus (e.g. pullulan). In addition to their natural properties such as biodegradability, biocompatibility and nontoxicity (Autissier et al., 2010; Mishra et al., 2011), these polymers can be tuned to gain appropriate biological or physical properties (Monteiro de Paula et al., 2015). Among these polymers, pullulan is a neutral, linear, non-immunogenic polysaccharide produced from starch fermentation by *Aureobasidium pullulans* (Leathers, 2003). It consists of glucose units linked through α-1,6- and α-1,4-glycosidic bonds (Shingel, 2004; Cheng et al., 2011). Pullulan can form a crosslinked network by the formation of intermolecular phosphate bonds (Lack et al., 2007). It has been widely used in the food, pharmaceutical and cosmetic industries for its functional properties including adhesiveness, film formability and enzymatically mediated degradability (Mishra et al., 2011). Of note, pullulan has also been used for the development of resorbable hydrogel-based drug delivery system (Thébaud et al., 2007; Purnama et al., 2015; Lu et al., 2017).

These converging data are likely to make pullulan a good candidate for the development of a bioactive DDS targeting IVD regenerative process. To allow sustained release of GDF-5 and TGF-β1, we herein encapsulated these growth factors in injectable pullulan microbeads (PMBs). In addition, to increase the retention of PMBs at the injection site within the degenerated IVD (Bertram et al., 2005; Zeng et al., 2015), we proposed that they could be associated to an injectable viscous solution that will undergo *in situ* crosslinking. To this end, we selected a self-setting hydrogel consisting in silanized hydroxypropyl methylcellulose (Si-HPMC) (Bourges et al., 2002) which has already been used *in vivo* notably for cell-based myocardium and cartilage regeneration strategies (Vinatier et al., 2007; Mathieu et al., 2012). The association of PMBs with Si-HPMC hydrogel will be called ‘biphasic system’, since such systems have been previously so-called in the relevant literature (Shimomura et al., 2014; Koushki et al., 2015; Puppi et al., 2016).

The objective of this work was thus to develop and characterize a biphasic system of PMBs dispersed into Si-HPMC as an injectable hydrogel-based drug delivery system for TGF-β1 and GDF-5, in order to stimulate cell-mediated IVD regenerative process.

## Experimental section

### Materials

All chemicals were used as received without any further purification. Dimethyl sulfoxide (DMSO, anhydrous ≥99.9%), pyridine (anhydrous, 99.8%), dibutyltin dilaurate (95%), Sodium hydroxide (NaOH ≥98%, pellets), fluorescein isothiocyanate isomer I (FITC ≥90%), Sodium trimetaphosphate (STMP ≥95%), sodium dodecyl sulfate (SDS ≥98.5%), potassium chloride (KCl 99.0–100.5%) and potassium phosphate monobasic (KH_2_PO_4_) were purchased from Sigma-Aldrich (St Louis, MO). Ethanol absolute NORMAPUR^®^ (≥ 99.8%), sodium chloride (NaCl), sodium dihydrogen phosphate dihydrate (NaH_2_PO_4_) were purchased from VWR (Fontenay-sous-Bois, France). Pullulan (200 000 g/mol) was purchased from Hayashibara Inc. (Okayama, Japan). Seringe SoftJect^®^ 3 mL, disposable HSW FINE-JECT^®^ needles 18 G (1.2 × 40 mm), 22 G (0.7 × 30 mm) and 23 G (0.6 × 30 mm) were purchased from Henke-Sass, Wolf GmbH (Tuttingen, Germany). Sterican needles (0.60 × 60 mm) were purchased from B Braun (Melsungen, Germany). Blood Transfusion Sets (ref: VH-22-EGA) were purchased from CareFusion (San Diego, CA).

Pierce BCA^TM^ Protein Assay Kit, Dulbecco's Modified Eagle Medium (DMEM), Ham's F-12 Nutrient Mix (F12), Penicillin/Streptomycin, HEPES 1 M, Gentamicin (50 mg/mL) and phosphate buffer saline (PBS) were purchased from ThermoFisher Scientific (Saint-Aubin, France). Fetal Bovine Serum (FBS, South America origin) was purchased from Dominique Dutscher (Brumath, France). Recombinant human GDF-5 and human TGF-β1 were obtained from Peprotech (Neuilly-sur-Seine, France).

### Pullulan microbeads formulation

#### FITC-labelled pullulan

A solution of FITC was prepared in anhydrous DMSO (10% w/v). In parallel, 1 g of pullulan was dissolved into 9 mL of anhydrous DMSO in a 15 mL glass tube topped by a septum. Few drops of pyridine, 1 mL of FITC solution and 20 mg of dibutyltin dilaurate were added in a glass tube and the reaction was allowed for 2 h at 95 °C. FITC-pullulan was purified in successive baths of cold 100% ethanol. It was then dissolved into distilled water and the solution was dialyzed at 4 °C for 3 days. Finally, FITC-pullulan was freeze-dried (CRYOTEC PILPA V7.52) and stored protected from light at room temperature until use.

#### FITC-pullulan solution

Non-labelled pullulan (6 g), combined with FITC-pullulan (10 mg), was dissolved into 20 mL of distilled water and stirred gently upon obtaining a homogeneous, viscous solution. This solution was stored at 4 °C, protected from light and used within the five following days.

#### Pullulan microbeads (PMBs) formulation

In a 150 mL beaker, 75 mL of rapeseed oil were stirred at room temperature. In parallel, in three different 3 mL syringes, (1) 2.5 g of FITC-pullulan solution, (2) 250 μL of 10 M NaOH solution and (3) 250 μL of STMP solution (90% w/v) were measured. Syringes (1) and (2) were connected using a luer-lock and the two solutions were mixed until reaching homogeneity. Syringe (3) was then connected and solutions were mixed vigorously. The resulting mixture was then injected in the oil phase under stirring at 450 rpm, using an 18 G needle. The water/oil emulsion was then placed at 37 °C, under constant stirring for 1h30 to allow crosslinking *via* the formation of phosphate bond between the STMP and the − OH groups on pullulan chains.

After pullulan crosslinking, PMBs were rinsed in successive baths of homemade phosphate buffer saline (PBS, pH 7.4), sodium dodecyl sulfate (SDS) solution and NaCl solution. PMBs were then filtered using a blood transfusion set with a mesh aperture of 200 μm, freeze-dried and stored at room temperature until use.

### Microscopy

Before freeze-drying, a sample of each PMBs batch was transferred to a glass slide for direct observation with a fluorescence microscope (DM IL LED Fluo - Leica, Germany). PMBs were also examined under a confocal microscope (Eclipse TE2000-E - Nikon, 94504 Champigny sur Marne, France) using a 488-nm laser and Z-stack images with a 3-μm step were obtained. Scanning Electronic Microscopy (SEM) was carried out using a LEO 1450VP microscope (Zeiss) by detecting secondary electrons at the following settings: 5 keV–13 pA. Samples were previously allowed to slowly dry at room temperature for a week and gold/palladium coated using a Desk III metal coater (Denton).

### PMBs characterization

#### Particle size analysis

A Mastersizer 3000 Laser (Malvern Instruments, UK) was used for particles size analysis. Approximately 30 mg of freeze-dried PMBs were re-hydrated in 5 mL PBS for 1 h. Experiments were performed in PBS and five measurements were done for each sample.

#### Mechanical compression

Mechanical properties of hydrated FITC-pullulan microbeads were investigated by subjecting them to a compressive force between 2 parallel plates for 30 s, in water at room temperature until reaching a deformation of 25% of their diameter (MicroSquisher^®^—CellScale Biomaterials Testing, Waterloo, Canada). The force (μN) and the displacement (μm) were measured using a micro-scale test system equipped with an integrated image analysis module. Results are expressed as the force applied versus the recorded displacement. The Young’s modulus was calculated according to the manufacturer recommendations and the standard expression: *E* = stress/strain = (*F*/A)/(Δl/l_0_) where *E* is the Young’s modulus; *F* is the force applied on a particle; *A* is the area through which the force is applied; Δl is the displacement and l_0_ is the initial diameter of the particle.

### Si-HPMC

Silanized hydroxypropyl methylcellulose (Si-HPMC) was prepared as previously described (Bourges et al., 2002; Fatimi et al., 2008). Briefly, HPMC was silanized using 3-glycidoxypropyltrime-thoxysilane (GPTMS). Si-HPMC was then dissolved into a basic media, at pH = 12.8 (sol). The gel of Si-HPMC was further obtained by lowering the pH with mixing it (2:1) with an acidic buffer (pH = 3.2). The final hydrogel has a pH of 7.4. For future references, ‘Si-HPMC hydrogel precursor’ will relate to the mix of the basic viscous solution with the acidic buffer and ‘Si-HPMC hydrogel’ will relate to the fully cross-linked hydrogel obtained after 10 days at 37 °C.

### PMBs/si-HPMC characterization

Freeze-dried PMBs were re-hydrated in PBS for 30 min and dispersed within Si-HPMC hydrogel precursor, in line with a previously described protocol (Buchtova et al., 2013; Henry et al., 2017). These biphasic systems were prepared with PMBs concentrations ranging from 0.3 to 1.6% (w/w).

#### Gel point

Immediately after mixing, PMBs/Si-HPMC hydrogel precursor was deposited onto a HAAKE MARS rheometer (Thermofisher, Saint-Aubin, France) plate and gel point was determined using a parallel plate sensor system (PP20 Ti geometry, gap size: *h* = 1 mm, temperature: 37 °C). A 20-step ramp with frequencies from 1 to 7 Hz was applied and G′ and G″ were recorded at each step. Gel point was determined by calculating the point at which tan δ = 1 for each frequencies.

#### Homogeneity

PMBs, with concentrations ranging from 0.3 to 1.6% (w/w), were dispersed within Si-HPMC hydrogel precursor either immediately after mixing or after 10, 20, or 30 minutes of crosslinking at room temperature. For clarity sake, only results after 30 minutes are presented here. After 10 days of cross-linking at 37 °C in a 12-well plate, top and bottom slices of cross-linked PMBs/Si-HPMC hydrogels, here an after referred as ‘biphasic system’, were observed with a fluorescence microscope. For all the subsequent experiments, PMBs were incorporated in the hydrogel precursor after 30 min of crosslinking at room temperature.

#### Injectability

For these experiments, 3 mL syringes containing the PMBs/Si-HPMC hydrogel precursor were connected to a 23 G needle and settled in the texture analyzer TA.HD*plus* (Texture Technologies, Hamilton, MA). Two milliliters injections were performed using a 500 kg-load cell with a trigger force of 5 g, a speed test of 2 mm/s and the applied force (N) was recorded.

#### Shear-stress

PMBs/Si-HPMC hydrogel precursor were prepared and deposited into 12-well plates and allowed to crosslink for 10 days at 37 °C. Shear-stress tests were performed on the biphasic system into the 12-well plates, using a HAAKE MARS rheometer (Thermofisher, Saint-Aubin, France) and a PP20 Ti geometry covered with sandpaper at 23 °C. This sandpaper was changed between each sample. A normal force of 1 N was applied and a 20 minute relaxation period was allowed. First, a frequency ramp was performed and we determined that at 1 Pa, storage modulus G’ was not affected by frequencies ranging from 0.01 to 4 Hz (data not shown). Shear stress ranging from 0.1 Pa to 3000 Pa was applied on the samples with a frequency set at 1 Hz. Storage modulus G’ and loss modulus G", as well as breaking stress, were recorded as a function of stress.

#### Compression

As for shear-stress experiments, biphasic systems were prepared and allowed to crosslink in 12-well plates for 10 days at 37 °C. Nevertheless, for compression experiments, biphasic systems were punched and removed from the 12-well plates. The obtained samples were 12 mm diameter large and 5 mm high. Compression was performed with the texture analyzer TA. HD*plus* (Texture Technologies, Hamilton, MA), using a 5-kg load cell with a trigger force of 1 g and a speed test of 0.01 mm/s. Run was allowed until complete destruction of the sample. The applied force (N) as a function on the displacement (mm) was recorded. The Young’s modulus was calculated as previously described in the mechanical characterization section.

### Growths factors loading release and bioactivity maintenance

#### Growth factors loading

The same amount of freeze-dried PMBs used for preparing 1.6% (w/w) concentrated biphasic system was used for each experimental condition exposed below. PMBs were suspended in 480 μL of 30 mM phosphate buffer pH 7. Growth factors loading was carried out by adding TGF-β1 or GDF-5 solution with a final concentration of 1, 2 and 4 μg/mL and a final volume of 500 μL. Controls were also performed (growth factor solution without PMBs and PMBs without growth factors). Loading was allowed for 24 h at 4 °C under rotary stirring at 24 rpm on a Mini LabRoller™ Dual Format Rotator (Labnet). Suspensions were then centrifuged for 10 min at 5000 rpm. Supernatants were removed and growth factor concentrations were determined using ELISA Duoset^®^ assay kits (RnDSystems - DY240 and DY583 for TGF-β1 and GDF-5, respectively). The impregnated growth factor amount and actual drug loading was calculated from the difference between initial and supernatant concentrations (data not shown).

#### In vitro release profiles

Centrifuged growth factor loaded PMBs were resuspended in 500 μL of 0.20 μm filtered PBS/BSA 1% (v/w) and stirred for 21 days at 37 °C. At selected times, suspensions were centrifuged for 10 minutes at 5000 rpm. Supernatants were collected, replaced by fresh PBS/BSA 1% (v/w) and kept at −80 °C for further experiments. Determination of growth factor concentrations in the supernatant were performed using ELISA Duoset^®^ assay kits (RnDSystems - DY240 and DY583 for TGF-β1 and GDF-5, respectively).

#### Release after dispersion into si-HPMC

Growth factor loaded PMBs were used to prepare biphasic systems. Controls were prepared using hydrogels loaded with growth factors without PMBs and PMBs loaded with growth factors without Si-HPMC hydrogels. Crosslinking was allowed for 3 h at 37 °C and 1 mL of PBS/BSA 1% (v/w) was added on the top of samples. Plates were then incubated at 37 °C on a vibrating platform shakers (Vibramax 100 - Heidolph Instruments GmbH&Co., Schwabach, Germany) for 28 days. At selected times, supernatants were collected, replaced by fresh PBS/BSA 1% (v/w) and kept at −80 °C for further experiments. Determination of growth factors concentrations in supernatants were performed using ELISA Duoset^®^ assay kits (RnDSystems - DY240 and DY583 for TGF-β1 and GDF-5, respectively).

#### Growth factors bioactivity after release

Mesenchymal stem/stromal cells isolated from human adipose tissue (hASC) were cultured using DMEM supplemented with 10% (v/v) FBS (Dominique Dutscher), 1% (v/v) Penicillin/Streptomycin (P/S) at 37 °C, 5% CO_2_ as extensively described previously (Merceron et al., 2012; Colombier et al., 2016). Cells were seeded in six-well plates at 10 000 cells/cm^2^ and allowed to proliferate until reaching approximately 80% confluence. They were then serum starved for 24 h using FBS-deprived above-described media before stimulation with released growth factors. Fresh solutions of both growth factors were prepared for control. Supernatants, with TGF-β1 or GDF-5 concentrations allowing to reach at least 1 ng/mL and 2 ng/mL, respectively, were deposited onto hASC and incubated 1 h at 37 °C. The medium was then removed and the plates were frozen with liquid nitrogen. For further western blot experiments, proteins were extracted on ice using a homemade RIPA buffer and assayed using the BCA^TM^ Protein Assay Kit. Protein migration was performed using 4–15% Criterion^TM^ TGX^TM^ precast gels (Bio-Rad), and proteins were transferred onto 0.2 μm PVDF membranes (Bio-Rad) using the Transblot^®^ Turbo^TM^ transfer system (Bio-Rad). As previously described (Colombier et al., 2016), P-Smad 1/5/8 (antibody 1/1000^th^ ref: 9511 S - Cell Signaling) was analyzed for cells stimulated with GDF-5. P-Smad 2/3 (antibody 1/1000^th^ ref: 3101 S - Cell Signaling) was analyzed for cells stimulated with TGF-β1. Β-actin (antibody 1/1000^th^ ref: A2228 - Sigma Aldrich) was analyzed for cells stimulated with both growth factors. All primary antibodies were revealed using a secondary antibody HRP-coupled (1/2000^th^ ref: 7074 S - Cell signaling) and the SuperSignal™ West Dura substrate (ThermoFisher Scientific) on the ChemiDoc^TM^ MP Imaging System (Bio-Rad) and Image Lab software.

### Statistics

All experiments were performed with replicate samples from independent conditions (*n* = 6 for PMBs mechanical characterization, *n* = 3 for gel point, *n* = 11 for injectability, *n* = 11 for shear stress analysis and *n* = 3 for compression stress analysis, *n* = 3 for loading and release). Data are given as the mean of independent replicates, and error bars represent the standard error of the mean. One-way ANOVA was calculated between different conditions, followed by post hoc Dunn’s multiple comparison test to determine significant differences (*p < .05, **p < .01, ***p < .001). All statistical analyzes were performed using Graphpad software.

## Results

### Pullulan microbeads formulation

**Figure 1.**
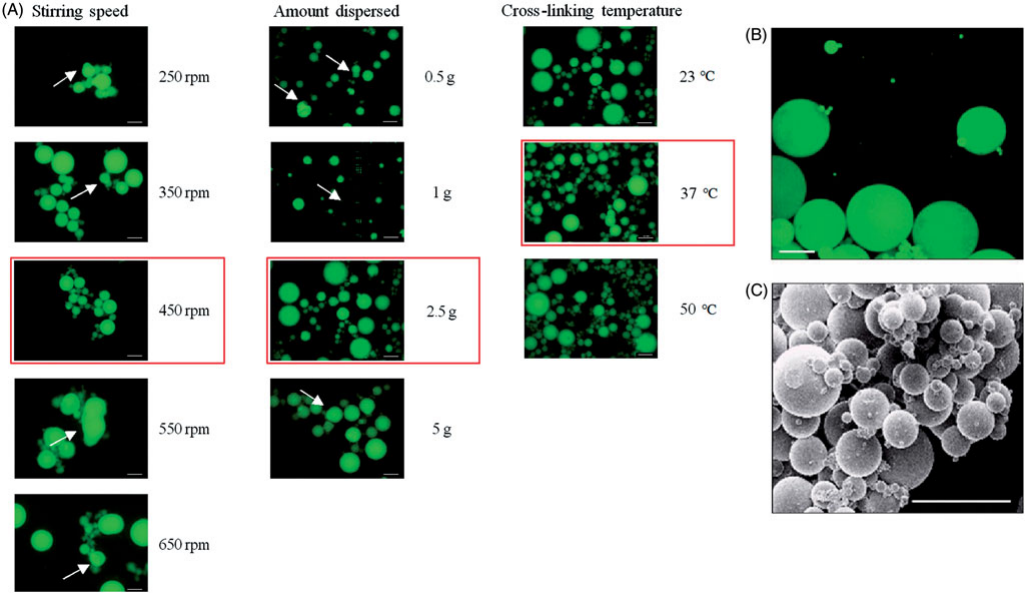
Pullulan microbeads optimization process and microscopy. PMBs observed with fluorescence microscopy. (A) PMBs were formulated according to a water-in-oil emulsion/crosslinking process with varying stirring speed, amount of pullulan solution dispersed in the oil phase and cross-linking temperature. Optimal conditions are highlighted in red; PMBs were then observed using (B) confocal microscopy and (C) Scanning Electronic Microscopy; Scale bar = 100 μm.

In a context of IVD injectable system, we aim at developing microcarriers that can be implanted through a 23 G needle (internal diameter = 318 μm) for growth factors sustained delivery. For this purpose, we optimized a previously described water-in-oil emulsion/crosslinking protocol (Bonnard et al., 2014; Aydogdu et al., 2016) for the formulation of PMBs by varying three parameters one after another. The formulated PMBs were subsequently observed with fluorescence microscopy (Figure 1(A)). We first varied the stirring speed of the oil phase from 250 rpm to 650 rpm. Unlike what we observed at other stirring speed, at 450 rpm no aggregate was detected and the microparticles were spherical. Then, we varied the amount of FITC-pullulan solution dispersed in the oil phase from 0.5 to 5 g. In this case, no aggregate was observed whatever the amount of pullulan dispersed. Nevertheless, PMBs surface frequently presented bumps and hollows, except for formulations with 2.5 g of FITC-pullulan solution dispersed. After that, the crosslinking temperature influence was studied and formulations were performed at 23 °C, 37 °C or 50 °C. Whereas at 50 °C, some bumps were observed on the PMBs, at 23 °C and 37 °C, spherical particles were obtained with a smooth surface. Consequently, we decided to further work at 37 °C. All subsequent experiments were thus performed using these optimal parameters: stirring speed of 450 rpm, 2.5 g of FITC-pullulan solution dispersed in the oil phase, crosslinking at 37 °C.

### Microscopy

Samples were prepared in the selected above-mentioned conditions. Confocal analysis showed on cross-sections (data not shown) and 3D reconstructions (Figure 1(B)) that dried PMBs were solid, without cavity, with a homogeneously dispersed fluorescence in the microparticles. Finally, scanning electronic microscopy observations demonstrated that PMBs exhibited a smooth surface and that the overall particles population showed an average diameter below 100 μm (Figure 1(C)).

### PMBs mechanical characterization

**Figure 2.**
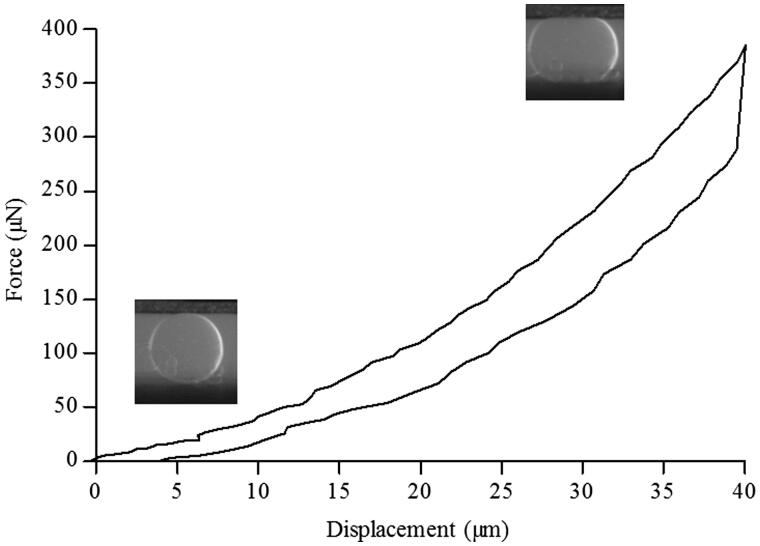
PMBs mechanical characterization. A hydrated single PMB was compressed between two plates using a MicroSquisher^®^ (CellScale). Force and displacement were recorded and a representative curve is presented.

Particle size analysis determined that 90% of the particles population had a diameter inferior to 184 μm (data not shown). We then studied hydrated single beads stiffness using a MicroSquisher^®^ (CellScale Biomaterials). The force (μN) and the displacement (μm) were measured using a micro-scale test system and a representative curve obtained with a single bead is presented Figure 2. We observed that, during compression, the applied force reached 400 μN for a final displacement of 40 μm. This compression phase was followed by a hysteresis loop, characteristic of a viscoelastic response of the material. Based on these data, we calculated a Young’s modulus of 57 ± 6 kPa.

### PMBs/si-HPMC characterization

Since we aim at injecting PMBs in patients with early degenerated lumbar disc, while avoiding leakage, we hypothesized that PMBs could be dispersed in a hydrogel scaffold which will crosslink *in situ*. We thus mixed PMBs with the Si-HPMC hydrogel precursor and characterized this biphasic system.

**Figure 3.**
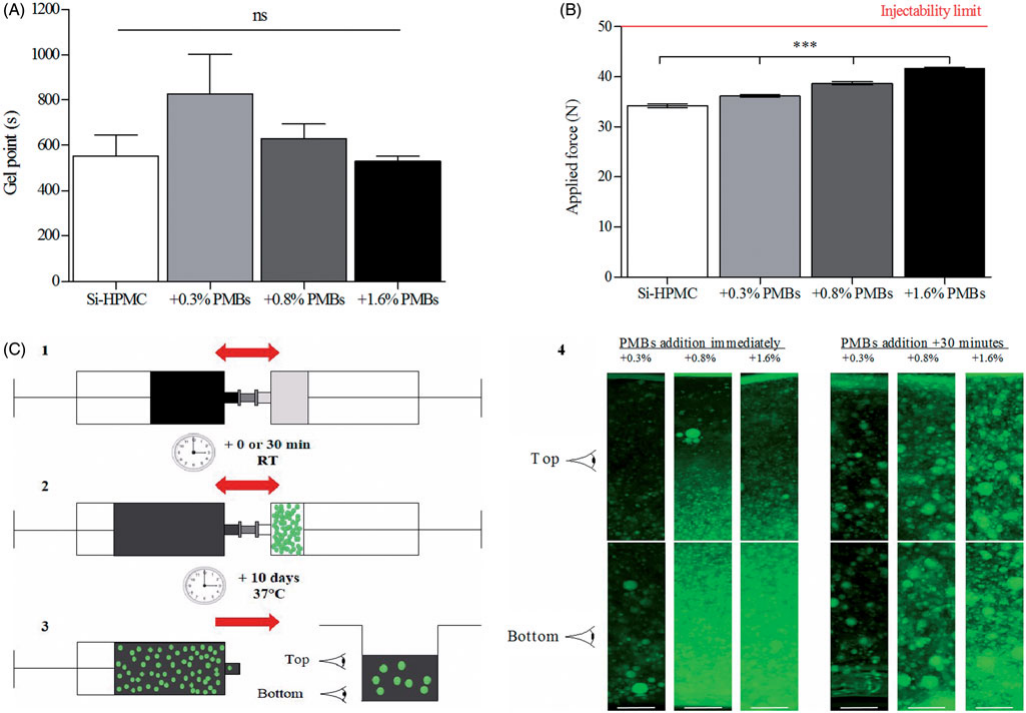
PMBs dispersion within Si-HPMC hydrogel precursor. PMBs were dispersed within the Si-HPMC hydrogel precursor and the influence on (A) its gel point and (B) its injectability were studied. These experiments were performed onto Si-HPMC alone or with PMBs at several concentrations ranging from 0.3 to 1.6% (w/w); (C) The PMBs dispersion in the Si-HPMC pre-hydrogel was also studied with the following steps: (1) Mix of basic viscous solution of Si-HPMC with acidic buffer, (2) Incorporation of PMBs in the pre-hydrogel immediately or after 30 min delay at room temperature. (3) PMB/Si-HPMC hydrogel was transferred into a 12-well plate. (4) After 10 days cross-linking at 37 °C top and bottom of hydrogel slices were observed; Scale bar =250 μm.

We first ensured that Si-HPMC hydrogel precursor was still able to crosslink when supplemented with PMBs at concentrations ranging from 0.3 to 1.6% (w/w) by studying the gel point (Figure 3(A)). G′ and G″ were recorded under shear stress with frequencies from 1 to 7 Hz and the gel point was determined for tan δ = 1 at each frequencies. The results presented in Figure 3(A) showed no significant modification of the time needed to reach the gel point of the Si-HPMC hydrogel precursor, whatever the amount of PMBs added, with a gel point of 8.8 ± 0.4 min and 9.2 ± 1.5 min for Si-HPMC hydrogel precursor alone or with the highest PMBs concentration.

The PMBs/Si-HPMC hydrogel precursor injectability was also studied. Syringes containing the PMBs/Si-HPMC hydrogel precursor were connected to a 23 G needle, injection was performed in the texture analyzer TA. HD*plus* ^®^ (Texture Technologies) and the applied force necessary to expel the mixture was recorded. Figure 3(B) shows a significant increase (from 34.3 N for the Si-HPMC hydrogel precursor alone to 41.6 N for the 1.6% (w/w) PMBs addition). Nevertheless, whatever the amount of PMBs added, the applied force remains under the injectability limit according to the norm ISO regulations/requirements 7886-1 which suggests a maximal injection force inferior to 50 N.

Having a system injectable and able to crosslink, we studied the distribution of the PMBs in the Si-HPMC hydrogel. Briefly, we dispersed PMBs within Si-HPMC hydrogel precursor either immediately or 30 min after mixing Si-HPMC basic viscous solution with the acidic buffer, at room temperature. After 10 days of crosslinking at 37 °C in a 12-well plate, top and bottom slices of PMBs loaded hydrogels were observed with a fluorescence microscope (Figure 3(C), step 4). We observed that when added immediately, the PMBs have a tendency to settle down at the bottom of the hydrogel, whatever the PMBs concentrations. Interestingly, for samples with a 30 minute delayed addition of PMBs, we observed a homogeneous dispersion of the PMBs in the top and the bottom part of the hydrogel, whatever the PMBs concentrations.

**Figure 4.**
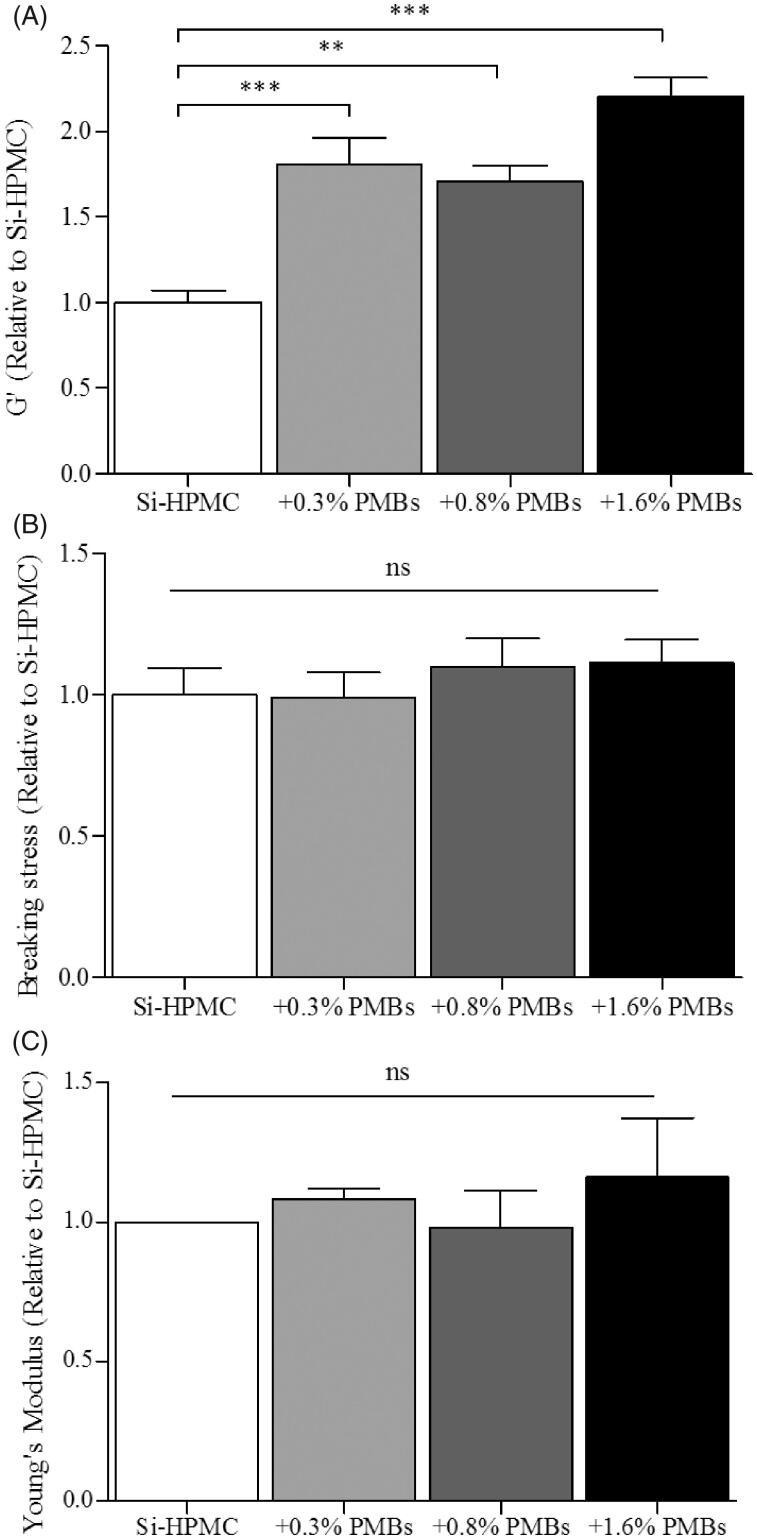
PMBs/Si-HPMC biphasic system mechanical characterization. Biphasic system was characterized after 10 days cross-linking at 37 °C, under (A-B) shear stress and (C) compression stress. Characterization was performed on control Si-HPMC or with adding concentration of PMBs ranging from 0.3% to 1.6% (w/w).

Finally, we studied the influence of PMBs addition on Si-HPMC mechanical properties under shear-stress and compression (Figure 4). Shear-stress analysis was performed onto biphasic systems as described in the experimental section and G’ as a function of stress, as well as breaking stress were recorded. Figure 4(A) shows that increasing amount of PMBs in the Si-HPMC hydrogel led to a significant 2 fold G′ increase (from 1168 ± 85 Pa for Si-HPMC alone to 2565 ± 136 Pa for Si-HPMC + 1.6% (w/w) PMBs). It is worth noting that despite the G’ increase, breaking stress of the hydrogel is not significantly affected by PMBs addition (Figure 4(B)). Finally, compression analysis was performed with a texture analyzer. A slight, although not significant, increase of the Young's modulus was noted when PMBs were added (Figure 4(C)).

### Growths factors loading and release

After having demonstrated our ability to manufacture and characterize PMBs either alone or embedded into Si-HPMC hydrogel, we then determined whether this biphasic system could be used for the sustained delivery of nucleopulpogenic growth factors. We were interested in TGF-β1 and GDF-5 loading/release profile, as we recently demonstrated (Colombier et al., 2016) the role of these two growth factors in the *in vitro* commitment and differentiation of hASC into nucleopulpogenic cells.

**Figure 5.**
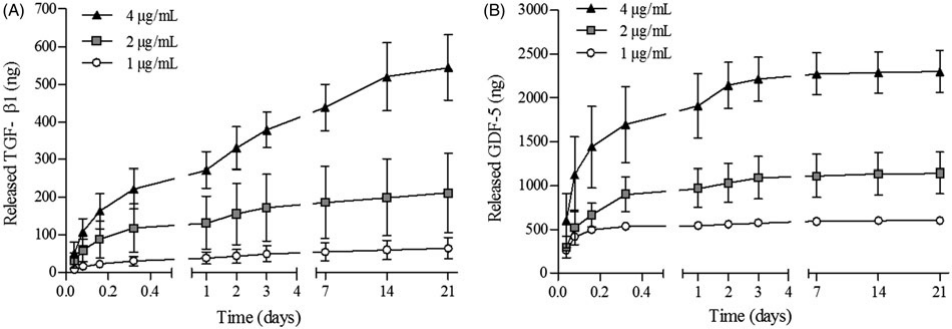
TGF-β1 and GDF-5 release kinetics from PMBs in PBS/BSA 1%. PMBs were loaded either with TGF-β1 or GDF-5 at 3 concentrations (1, 2 or 4 μg/mL). Release was performed in PBS/BSA 1% at 37 °C. At specific time point, supernatants were retrieved and analyzed with ELISA. Results are expressed as the cumulative amount of (A) released TGF-β1 and (B) released GDF-5 as a function of time.

Freeze-dried PMBs were thus loaded with growth factors and release experiments were then performed in PBS/BSA 1% (v/w), at pH 7.2, 37 °C. Growth factors assays on supernatants after up to 21 days release were performed as described in the experimental section. The loading efficiency of both growth factors was 100% as we were not able to detect them in the supernatants collected after loading (data not shown). Release profiles of TGF-β1 and GDF-5 are presented on Figure 5(A) and (B), respectively. The release rate decreased during the 21 days, whatever the initial amount of growth factor loaded in PMBs, from 50 ng/h to 0.15 ng/h and from 604 ng/h to 0.6 ng/h during the first hour and the last 7 days, for TGF- β1 and GDF-5, respectively (experiments performed with growth factors initial concentrations of 4 μg/mL). In addition, we observed that, while released TGF-β1 reached approximately 40% of the initially loaded amount, GDF-5 was entirely released from the PMBs, whatever the initial impregnated amount.

**Figure 6.**
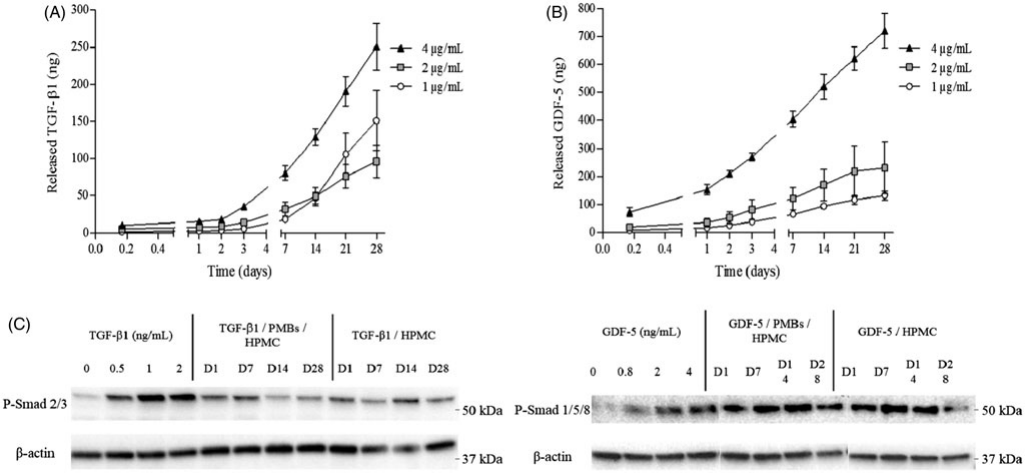
TGF-β1 and GDF-5 release kinetics from PMBs/Si-HPMC biphasic system and biological activity. PMBs were loaded either with TGF-β1 or GDF-5 at 3 concentrations (1, 2 or 4 μg/mL). PMBs were associated with Si-HPMC hydrogel precursor and after 3 h of crosslinking at 37 °C, release was performed by adding PBS/BSA 1% on the top of hydrogels. At specific time points supernatants were retrieved and analyzed with ELISA. Results are expressed as the cumulative amount of (A) released TGF-β1 and (B) released GDF-5 as a function of time. (C) Supernatants were further deposited onto hASC during 1 h for exploring the released TGF-β1 and GDF-5 bioactivity maintenance by western blot.

In parallel, growth factor loaded biphasic systems (PMBs/Si-HPMC) were prepared as described in the experimental section. Crosslinking was allowed for 3 h at 37 °C and 1 mL of PBS/BSA 1% (v/w) was added on the top of the hydrogel and the release was performed for 28 days (Figure 6(A and B)). Interestingly, we observed a delayed release of the growth factors in these working conditions. Indeed, while the maximal release rates were observed within the first 24 h for PMBs in PBS/BSA 1% (v/w), when the PMBs were dispersed within Si-HPMC hydrogel, only small amounts of growth factors were released in this time period (0.64 and 6.36 ng/h for TGF-β1 and GDF-5, respectively). Released amount increased continuously until 28 days, although release rates did not increase in this period of time (0.35 and 0.59 ng/h for TGF-β1 and GDF-5, respectively).

Finally, the maintenance of growth factors bioactivity after release was studied. It has been shown that Smad 1/5/8 and Smad 2/3, intracellular signaling proteins, are the main effectors activated upon GDF-5 and TGF-β1 stimulation (Colombier et al., 2016). Herein, we were able to demonstrate the phosphorylation of both Smad 1/5/8 and Smad 2/3 after stimulation of hASC with early (1-day) as well as later released (up to 28 days) TGF-β1 and GDF-5, respectively (Figure 6(C)), confirming that growth factors released from PMBs through Si-HPMC hydrogel network were still bioactive.

## Discussion

The objective of the present work was to develop an injectable hydrogel-based biphasic delivery system able to sustainably deliver TGF-β1 and GDF-5 in a damaged IVD, with the ultimate goal of stimulating cell-mediated IVD regenerative process. Therefore, we hypothesized that TGF-β1 and GDF-5 loaded PMBs, dispersed into Si-HPMC hydrogel, would provide appropriate injectability, release and biological properties.

Since its first discovery in 1938 by Bauer, pullulan has been widely studied and its properties such as high water solubility, lack of toxicity, lack of immunogenicity, biocompatibility and biodegradability were highlighted (Autissier et al., 2010; Mishra et al., 2011). In addition, its physicochemical properties can be tuned to lower its solubility, introduce charges or add functionalities (Masuda et al., 2001; Prajapati et al., 2013). Synthetic polymers can also be grafted to pullulan to form stimuli-sensitives particles (Fundueanu et al., 2008; Morimoto et al., 2013; Nishimura et al., 2015), or bioconjugates (Suginoshita et al., 2002). In line with these advantages, pullulan have raised growing interest for biomedical applications and its use as a drug carrier for controlled release has been investigated (Leathers, 2003), To this end, pullulan can be formulated in micro- or nanoparticles either by self-assembling (Jeong et al., 2006; Wang et al., 2014) or with using a crosslinker (Lack et al., 2004). Herein, pullulan microbeads were formulated using a water-in-oil emulsion/reticulation process involving sodium trimethaphosphate (STMP) as a crosslinker. This crosslinker is of particular interest as it is nontoxic and commonly used in food industries (Woo & Seib, 1997). Noteworthy, surfactants are commonly used for improving emulsion stability (Kim et al., 2010; Aydogdu et al., 2016). Owing to their known potential toxicity (Yang et al., 2015), our formulation process was achieved without any surfactant addition.

With the aim of developing a new strategy for the IVD regenerative medicine, we considered the formulation of an intradiscal injectable biphasic system for the delivery of nucleopulpogenic growth factors, TGF-β1 and GDF-5 (Colombier et al., 2016). To increase PMBs retention after intradiscal injection, we hypothesized that they could be dispersed within an injectable hydrogel solution able to crosslink *in situ.* Si-HPMC is of particular interest, as it is a self-setting polymer whose crosslinking is triggered by pH (Bourges et al., 2002), unlike some other polymers which requires external or internal stimuli for crosslinking (Qiu & Park, 2012; Heo et al., 2016). Considering this specific property, Si-HPMC hydrogel has already been demonstrated to be a relevant scaffold in tissue engineering for cell-based myocardium or joint cartilage regeneration (Vinatier et al., 2009; Mathieu et al., 2012; Portron et al., 2013). Our data demonstrated that it was possible to disperse PMBs within Si-HPMC hydrogel precursor without impairing its gelation capacity neither than its injectability through a 23 G needle (Figure 3). Interestingly, it has been established *in vivo*, that a needle puncture in IVD may cause its degeneration *via* a direct mechanical degradation (*Annulus fibrosus* damage or *Nucleus pulposus* depressurization), and/or through not yet clearly defined biological mechanisms (Michalek et al., 2010; Martin et al., 2013). Elliott et al. (2008) conducted a literature review of animal models and showed that these alterations depend of the needle diameter to disc height ratio, with significant changes observed for ratios over 40% (Elliott et al., 2008). However, it is worth noting that needles as large as 18 G are used for patient disc injections for diagnosis or treatment, corresponding to a needle diameter to disc height ratio of less than 10% (Peh, 2005). Injecting PMBs/Si-HPMC through a 23 G needle should thus allow to target the degenerative process of NP while avoiding deleterious effects on IVD integrity. This undoubtedly strengthens the transferable aspect of our IVD bioactive delivery system towards clinical application. In addition, whether the intradiscal injection of a hydrogel in a degenerated IVD may contribute to restore hydration of the altered tissue, deserves to be further addressed in preclinical experiments, as it could create a 3 D micro-environment that could favor cell invasion and proliferation (Balkovec et al., 2016).

In a first set of experiments, we demonstrated the homogeneous dispersion of PMBs in Si-HPMC hydrogel precursor and their injectability. Then, for the first time, compressive mechanical properties of PMBs alone was studied using a MicroSquisher^®^ and PMBs Young’ Modulus was determined at 57 ± 6 kPa. Of note, Si-HPMC hydrogel alone exhibits a Young’s Modulus of approximately 1 kPa. Hence, biphasic systems containing homogeneously dispersed PMBs at concentrations ranging from 0.3% to 1.6% (w/w) were prepared and their mechanical properties were studied to determine whether PMBs addition would alter Si-HPMC hydrogel properties or not. Results obtained from the frequency ramp highlighted that G’ storage modulus of Si-HPMC hydrogel was not affected at frequencies ranging from 0.01 to 4 Hz (data not shown). In addition, the G’ values superior to the G″ values and the behavior of both moduli being frequency-independent indicates the gel-like behavior of Si-HPMC hydrogel. During shear-stress experiments we observed that, while the storage modulus (G’) was significantly increased whatever the amount of PMBs added (Figure 4(A)), we did not observe any significant modification of either the breaking stress or the Young’s modulus (Figure 4(B and C)). Within shear stress experiments, the storage modulus evolution reflects an increase in the elastic portion of the biphasic system. To further explain these observations, our hypothesis is that Si-HPMC hydrogel would benefit from the adding effect of the PMBs. At the studied concentrations, PMBs would not form a percolating network, since to reach this threshold for spherical particles, a volumic fraction of 0.3 is needed (Brandon & Kaplan, 2008) and we reach only 0.14 with the highest PMBs concentration. Thus, PMBs would rather create local concentrations of Si-HPMC thereby increasing its resistance towards shear stress at small deformations. Nevertheless, for larger deformations (e.g. breaking stress Figure 4(B)), the adding effect and interactions of the PMBs with the Si-HPMC hydrogel network were not sufficient to increase the breaking stress. Finally, the absence of Young’s modulus modifications can be explained by the polymer chains and PMBs reorganization during the slow compression (0.01 mm/s), leading to the gel rupture before any detectable effect of the PMBs. Altogether these results indicate that PMBs addition in the Si-HPMC hydrogel precursor has little impact on the fully crosslinked hydrogel mechanical properties, in our working conditions.

In the last part of this study, we explored the PMBs and PMBs/Si-HPMC capacity to sustainably deliver two growth factors, TGF-β1 and GDF-5. The loaded amounts were chosen according to a previously conducted work, where the optimal conditions for hASC differentiation towards NP cells were investigated (Colombier et al., 2016). Interestingly, we could demonstrate that these growth factors were released from PMBs alone for 21 days (Figure 5) and for up to 28 days when PMBs were dispersed within Si-HPMC hydrogel (Figure 6(A and B)). We can hypothesize that this release would probably continue even after 28 days. Regarding PMBs alone, release kinetics showed a fast growth factor release during the first 3 days, up to 50 and 604 ng/h, representing over 75% and 95% for TGF-β1 and GDF-5, respectively. This difference may be explained by a faster diffusion rate of the GDF-5 as compared to the TGF-β1. These growth factors are typically presented with molecular weights of 13 and 25 kDa, respectively. Interestingly, we obtained from our shear stress measurements a pullulan network mesh size in the PMBs in the range of 5 nm, calculated from the following Flory equation,

$$\xi =\sqrt[{3}]{\frac{k_{B}T}{G\text{'}}}$$

where *ξ* is the mesh size, G’ the storage modulus, k_B_ the Boltzman constant and T the temperature (K) (Flory, 1953). This mesh size appears similar to the GDF-5 size, allowing its faster diffusion than that of TGF- β1. The observed release rates were much lower in the following days, especially for GDF-5. Interestingly, Kim et al. (2010), demonstrated that release rates of a loaded protein from microparticles can be easily modulated with surface coating. Indeed, they coated alginate microparticles with increasing chitosan amounts, from 1 to 3% (w/v) (Kim et al., 2010). Accordingly, they observed reduced release rates with increasing the chitosan coating amount and related these observations to a densification of the polymeric network that would impede molecules from diffusing. In our study, when the PMBs were dispersed within Si-HPMC, the release was delayed and almost no growth factor could be detected in the supernatant during the first 24 h, with release rates of 0.64 and 6.36 ng/h for TGF-β1 and GDF-5, respectively. Thereafter, the released amount continuously increased until day 28. In all conditions, we observed that an increased amount of loaded growth factors led to a higher released amount. These results are in accordance with otherwise published studies that demonstrated a slower growth factor release when using a biphasic system (Holland et al., 2004) with a reduced initial burst and an overall prolonged release for up to 14 days (Nath et al., 2013). Additionally, Holland et al. (2005) performed release experiments using a biphasic system where they loaded two growth factors either in microparticles embedded in a hydrogel matrix and/or in the matrix itself. They thus demonstrated that it is possible to modulate the release profiles by creating a dual delivery system that could be applicable to ours (Holland et al., 2005).

In parallel, Wenk et al. (2009) demonstrated that bioactivity of the released molecule from a biphasic system was maintained for a longer period of time (Wenk et al., 2009). With our biphasic system, released GDF-5 and TGF-β1 were used to stimulate hASC in culture. We were able to demonstrate that 28-days released GDF-5 and TGF-β1 could activate intracellular signaling cascades as evidenced by the phosphorylation of Smad proteins. Interestingly, we previously showed that Smad 1/5/8 and Smad 2/3 pathways are essential to the differentiation of hASC. Indeed, Smad 2/3 pathway was demonstrated to be involved in the early commitment of the cells whereas the Smad 1/5/8 pathway was required for the maturation of differentiating cells (Colombier et al., 2016). Thus, by showing the ability of the released growth factors to induce the phosphorylation of the Smad 1/5/8 and Smad 2/3 pathways, we demonstrated their biological activity maintenance for up to 28 days, in our working conditions. These finding suggest that this hydrogel-based biphasic system allows the delivery of bioactive growth factors that, in turns, could stimulate cell-mediated IVD regenerative process. With regards to IVD regenerative medicine, this delivery system could also be used to tackle inflammation, notably triggered by TNF-α and some interleukins *via* the up regulation of MMPs and aggrecanases (Kepler et al., 2013; Wang et al., 2015). Indeed, TGF- β contributes to maintaining the expression levels of connective tissue growth factor (CCN2), which was demonstrated to suppress the inductive effect of IL-1β on catabolic genes (Tran et al., 2014). Additionally, GDF-5, down-regulated by IL-1β and TNF-α, has been shown to favor cell proliferation and stimulate the synthesis of proteoglycan and type II collagen, thus demonstrating its anabolic activity (Chujo et al., 2006).

Additionally, it has been described that stem/progenitor cell chemo-attraction from surrounding tissues occur in degenerated IVD (Illien-Jünger et al., 2012; Pattappa et al., 2014). This is of particular interest since, under GDF-5 and TGF-β1 treatment for 28 days, stem cells are able to differentiate towards NP cells, while producing an extracellular matrix comparable with that of the native NP (Colombier et al., 2016).

## Conclusions

Herein, we demonstrated the capacity of both PMBs alone and PMBs/Si-HPMC biphasic system to deliver growth factors *in vitro* for a sustained period of time. PMBs mechanical properties and their addition impact onto Si-HPMC hydrogel were explored. We observed that it was possible to disperse PMBs within Si-HPMC hydrogel precursor without impairing its gelation capacity neither than its injectability through a 23 G needle. Additionally, PMBs/Si-HPMC biphasic system showed a gel-like behavior and only little impact of PMBs addition on mechanical properties were highlighted. This finding suggest that PMBs would probably not create strong interactions with Si-HPMC hydrogel network, neither by crosslinking effect nor PMBs percolation. Additionally, the adsorption/release of two growth factors, TGF-β1 and GDF-5, was studied and a sustained delivery for up to 28 days was obtained. The maintenance of the biological activity of the released growth factors from PMBs was also shown. It is worth pointing out that the kinetic release of growth factors, up to 28 days, and their biological activity maintenance, appears to be compatible with a previously described hASC differentiation protocol towards NP cells (Colombier et al., 2016). These data suggest that PMBs/Si-HPMC biphasic system may be a promising candidate for the development of an innovative bioactive drug delivery system. Further experiments will determine whether growth factor loaded-PMBs/Si-HPMC biphasic system could be a suitable system for IVD regenerative medicine. In this view, we recently developed animal models of degenerative disc disease (Fusellier et al., 2016) in which preclinical relevance of our biphasic system could be assessed. If successful, our data may offer new therapeutic window in the treatment of degenerative disc disease, which remains a huge unmet medical need.
